# 13-Butoxyberberine Bromide Inhibits Migration and Invasion in Skin Cancer A431 Cells

**DOI:** 10.3390/molecules28030991

**Published:** 2023-01-19

**Authors:** Phuriwat Laomethakorn, Malatee Tayeh, Siritron Samosorn, Chantra Tananyuthawongse, Ramida Watanapokasin

**Affiliations:** 1 Department of Biochemistry, Faculty of Medicine, Srinakharinwirot University, Bangkok 10110, Thailand; 2 School of Allied Health Sciences, Walailak University, Nakhon Si Thammarat 80161, Thailand; 3 Hematology and Transfusion Science Research Center, Walailak University, Nakhon Si Thammarat 80161, Thailand; 4 Department of Chemistry, Faculty of Science, Srinakharinwirot University, Bangkok 10110, Thailand

**Keywords:** 13-butoxyberberine bromide, metastasis, skin cancer, MMPs, TIMPs, EGFR

## Abstract

Cancer metastasis is the primary cause of cancer morbidity and mortality. Anti-metastasis mechanism of skin cancer by 13-butoxyberberine bromide, a novel berberine derivative, has not yet been reported. This study investigated the effects of 13-butoxyberberine bromide on migration and invasion of skin cancer A431 cells. The cytotoxicity of 13-butoxyberberine bromide was determined by MTT assay. The effect of 13-butoxyberberine bromide on cell migration and invasion were examined using a wound-healing assay, transwell migration assay, and transwell invasion assay, respectively. The cell adhesion ability was determined by an adhesion assay. Protein expressions that play important roles in cancer migration and invasion were evaluated by Western blot analysis. The results showed that 13-butoxyberberine bromide effectively inhibited cell migration, invasion, and adhesion in A431 cells. Interestingly, 13-butoxyberberine bromide was more effective for cell migration inhibition than berberine. In addition, 13-butoxyberberine bromide showed anti-migration and anti-invasion effects by down-regulated MMP-2 and MMP-9 expression and up-regulated TIMP-1 and TIMP-2 expression in A431 cells. Moreover, pretreatment with 13-butoxyberberine bromide significantly inhibited EGF-induced cell migration and p-EGFR, ERK, p-ERK, STAT3, and p-STAT3 expressions in A431 cells at lower concentrations when compared with the berberine. These findings indicated that 13-butoxyberberine bromide could be further developed as an anticancer agent.

## 1. Introduction

Cancer metastasis is the leading cause of cancer-related death. Most cancer deaths are caused by cancer metastasis, not the primary tumor [1,2]. Metastasis is a multistep process that includes cell invasion, cancer cells entering blood vessels, leaving the circulatory system, and colonizing distant organs [3].

Generally, metastatic processes implicate the invasion and digestion of basement membrane through the release of proteolytic enzymes, matrix metalloproteinases (MMPs), by cancer cells. In particular, MMP-2 and MMP-9 play an important role in altering the extracellular matrix (ECM) via ECM protein digestion in the basement membrane. Due to the proteolytic action of MMPs, the altered ECM contributes to the movement and spreading of cancer cells to distant organs through the circulatory and lymphatic systems. Then, cancer cells divide and colonize to become a secondary tumor [4]. The active form of MMPs is inhibited by tissue inhibitors of metalloproteinases (TIMPs) such as TIMP-1 and TIMP-2 [5]. The net balance between MMPs and TIMPs determines the proteolytic potential of the tumor. A previous study showed that high levels of TIMPs could reduce metastasis, while low levels of TIMP correlated with cancer metastasis [6].

Epidermal growth factor receptor (EGFR) is a member of the receptor tyrosine kinases (RTKs) family, which is an important targeted therapy in cancer treatment [7]. Previous studies showed that an increased EGFR expression was related to many cancer types [8]. When EGFR was activated, proliferation, differentiation, transformation, angiogenesis, migration, and survival of cancer cells were induced [9]. Phosphorylation of EGFR activates the downstream components including ERK, PI3K/Akt, and STAT signaling pathways [10], which play a crucial role in cancer metastasis [11]. In addition, several studies reported that the EGFR signaling pathway also regulated MMP expression [12,13]. Therefore, it has been suggested that control of MMPs, TIMPs, and EGFR signaling pathway expression is a potential strategy for the improvement of anti-metastatic drugs.

Berberine, a plant isoquinoline alkaloid with a history in Ayurvedic and Chinese medication [14] and a yellow color [15], is isolated from many plants including *Coscinium fenestratum* and *Arcangelisia flava* [16], and is abundantly found in Berberidaceae families such as *Berberis vulgaris* (barberry), *Berberis aquifolium* (Oregon grape), and an Indian species *Berberis aristata* (tree turmeric) [17]. It is currently known that berberine has many pharmacological activities including antioxidant, anti-inflammation, antibacterial, anticholinergic, antihypertensive, and anticancer activities [18,19]. A previous study suggested that berberine had an anti-metastatic effect on cancer cells through down-regulated expression of MMP-2 and MMP-9 [20]. Berberine also suppressed epithelial–mesenchymal transition in mouse melanoma B16 cells [21] and inhibited the metastatic potential of melanoma cells [22]. In addition, it has been reported that berberine suppressed the EGFR signaling pathway [23]. There are many reports on the anti-metastatic effect of berberine, however, there are limited data about an inhibitory effect of berberine derivatives on metastasis. A previous report found that 13-butoxyberberine bromide was highly effective against several cancer cells. Moreover, it was more effective than berberine and the anticancer drugs ellipticine and doxorubicin [24].

Therefore, in this study, we examined the effects of 13-butoxyberberine bromide on migration and invasion and the underlying mechanisms of the EGFR-overexpressed skin cancer cell line A431, and demonstrated that 13-butoxyberberine bromide was more effective than berberine. The obtained information from this research can be used to develop berberine derivatives for cancer treatment in the future.

## 2. Results

### 2.1. The Cytotoxic Effect of 13-Butoxyberberine Bromide and Berberine on A431 Cells

**Figure 1:**
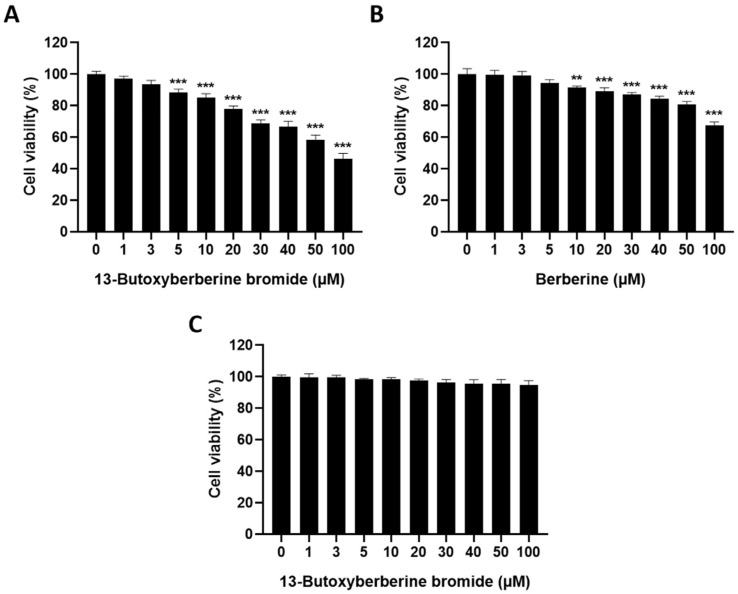
Effect of 13-butoxyberberine bromide and berberine on cell viability in A431 and HaCat cells. Cells were incubated with 13-butoxyberberine bromide and berberine at different concentrations (0–100 µM). The histograms present the percentage of A431 cell viability after treatment with 13-butoxyberberine bromide (**A**) and berberine (**B**), and HaCat cell viability after treatment with 13-butoxyberberine bromide (**C**), compared with the control group (0 = 0.5% DMSO). Data were expressed as mean ± SD (*n* = 3). ** *p* < 0.01 and *** *p* < 0.001; significant difference from the control group.

In this study, we examined the effect of 13-butoxyberberine bromide on skin cancer A431 cells compared to berberine. The cytotoxic effect of 13-butoxyberberine bromide and berberine on human skin cancer A431 cells was determined using an MTT assay. The results indicated that 13-butoxyberberine bromide reduced cell viability of A431 cells in a dose-dependent manner (Figure 1A). Moreover, 13-butoxyberberine bromide had a higher cell growth inhibitory effect when compared with the berberine (Figure 1B) and was non-toxic to human keratinocyte HaCat cells (Figure 1C). The sub-toxic concentrations of 13-butoxyberberine bromide (1, 3, and 5 µM) and berberine (10, 30, and 50 µM) were chosen for further experiments.

### 2.2. 13-Butoxyberberine Bromide Inhibited A431 Cell Migration More Effectively Than Berberine

**Figure 2:**
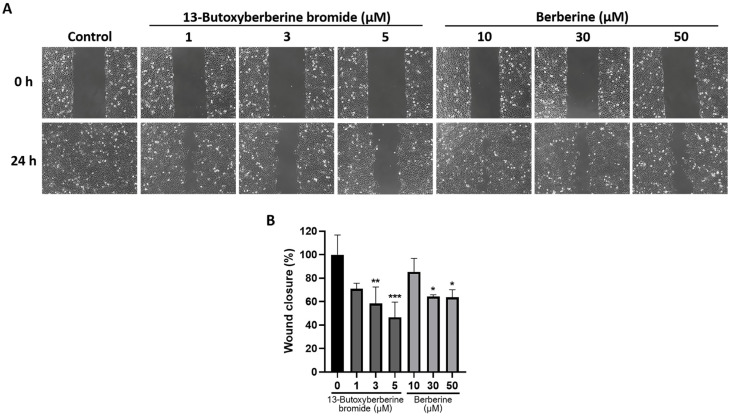
Inhibitory effect of 13-butoxyberberine bromide and berberine on wound healing in A431 cells. Cells were scratched and incubated with different concentrations of 13-butoxyberberine bromide and berberine before investigating the wound areas. (**A**) Wound distance was observed under an inverted microscope (100× magnification). (**B**) The histogram presents the wound closure percentage after incubation with 13-butoxyberberine bromide and berberine. Data were expressed as mean ± SD (*n* = 3). * *p* < 0.05, ** *p* < 0.01 and *** *p* < 0.001; significant difference from the control group.

The effect of 13-butoxyberberine bromide and berberine on cell migration in A431 cells was examined using a wound-healing assay. The results indicated that both 13-butoxyberberine bromide and berberine significantly suppressed cell migration in A431 cells compared to the control group (0.5% DMSO) (Figure 2). Interestingly, at concentrations of 1, 3, and 5 µM, 13-butoxyberberine bromide inhibited wound closure by 29.0%, 41.6%, and 53.4%, respectively, while 10, 30, and 50 µM berberine inhibited wound closure by 14.6%, 35.6%, and 36.4%, respectively. These results suggested that 13-butoxyberberine bromide showed more effective migration inhibition on A431 cells than berberine.

### 2.3. Effect of 13-Butoxyberberine Bromide on A431 Cell Migration using Transwell Migration Assay

**Figure 3:**
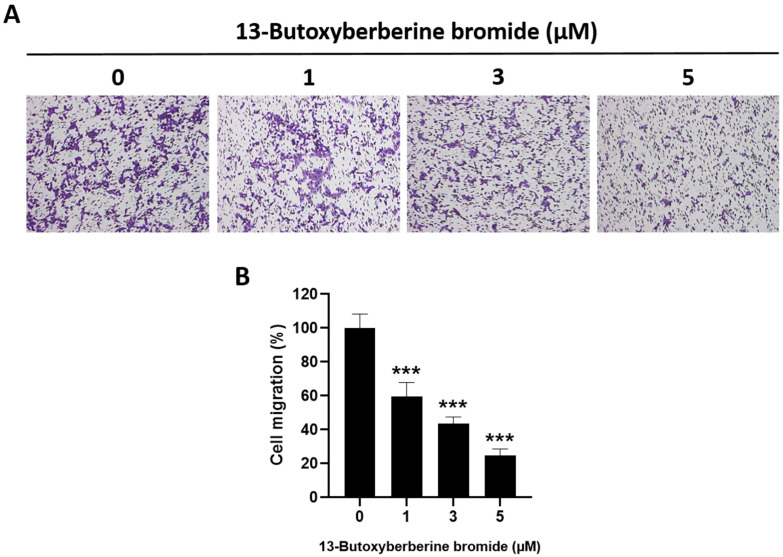
Inhibitory effect of 13-butoxyberberine bromide on cell migration in A431 cells. (**A**) Morphology of migrated A431 cells after staining with crystal violet under an inverted microscope (100× magnification). (**B**) The histogram shows percentage of cell migration after incubation with 13-butoxyberberine bromide. Data were expressed as mean ± SD (*n* = 3). *** *p* < 0.001; significant difference from the control group.

The effect of 13-butoxyberberine bromide on A431 cell migration was examined using a transwell migration assay. The results suggested that 13-butoxyberberine bromide significantly suppressed cell migration in A431 cells compared to the control group (0.5% DMSO) (Figure 3A). Cell migration in A431 cells was suppressed approximately 40.4%, 56.6%, and 75.4% after treatment with 1, 3, and 5 µM of 13-butoxyberberine bromide, respectively (Figure 3B). These results suggested that 13-butoxyberberine bromide could inhibit cell migration in A431 cells.

### 2.4. Effect of 13-Butoxyberberine Bromide on A431 Cell Invasion by Transwell Invasion Assay

**Figure 4:**
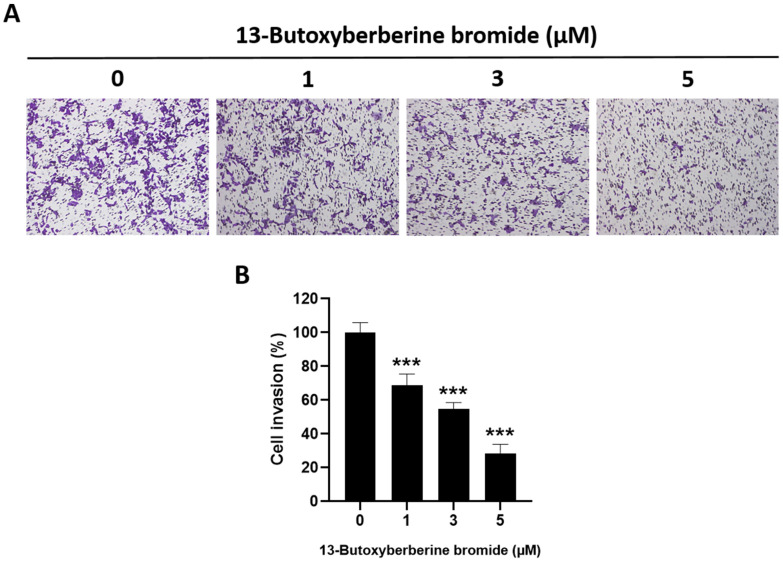
Inhibitory effect of 13-butoxyberberine bromide on cell invasion in A431 cells. (**A**) Morphology of invaded A431 cells after staining with crystal violet under an inverted microscope (100× magnification). (**B**) The histogram shows percentage of cell invasion after incubation with 13-butoxyberberine bromide. Data were expressed as mean ± SD (*n* = 3). *** *p* < 0.001; significant difference from the control group.

The effect of 13-butoxyberberine bromide on cell invasion in A431 cells was determined using a transwell invasion assay. The results indicated that 13-butoxyberberine bromide suppressed A431 cell invasion across the Matrigel-coated membrane filter compared to the control group (0.5% DMSO) (Figure 4A). Cell invasion in A431 cells was reduced by 31.3%, 45.2%, and 71.5% after treatment with 1, 3, and 5 µM of 13-butoxyberberine bromide, respectively (Figure 4B). These results suggested that 13-butoxyberberine bromide significantly inhibited A431 cell invasion.

### 2.5. Effect of 13-Butoxyberberine Bromide on Cell Adhesion in A431 Cells

**Figure 5:**
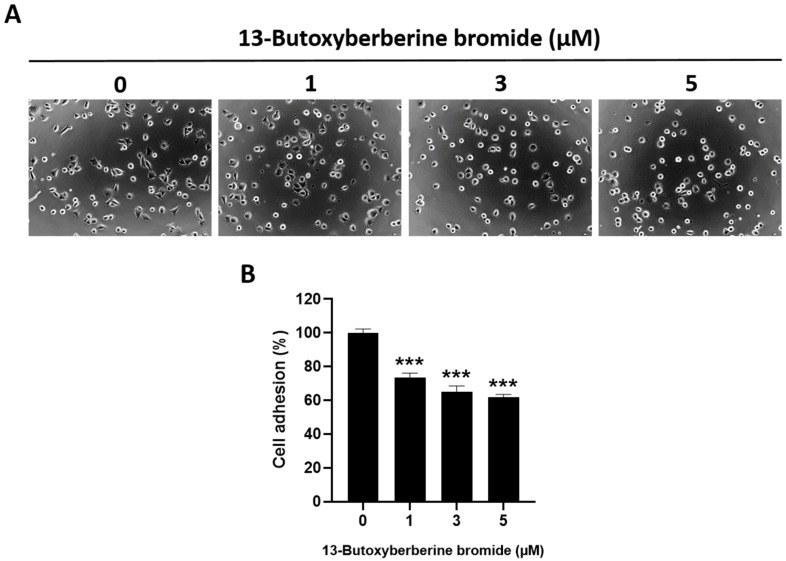
Inhibitory effect of 13-butoxyberberine bromide on cell adhesion in A431 cells. (**A**) Morphology of A431 cells after treatment with 13-butoxyberberine bromide cultured in Matrigel-coated plate (200× magnification). (**B**) The histogram shows percentage of cell adhesion after incubation with 13-butoxyberberine bromide. Data were expressed as mean ± SD (*n* = 3). *** *p* < 0.001; significant difference from the control group.

The effect of 13-butoxyberberine bromide on cell adhesion in A431 cells was examined using an MTT assay. The results suggested that 13-butoxyberberine bromide reduced the adhesion of A431 cells to the Matrigel-coated plate compared to the control group (0.5% DMSO) (Figure 5A). The adhesion ability of A431 cells was reduced by 26.6%, 34.9%, and 38.2% after treatment with 1, 3, and 5 µM of 13-butoxyberberine bromide, respectively (Figure 5B). These results indicated that 13-butoxyberberine bromide could reduce cell adhesion ability in A431 cells.

### 2.6. Effect of 13-Butoxyberberine Bromide on MMPs and TIMPs Expressions in A431 Cells

**Figure 6:**
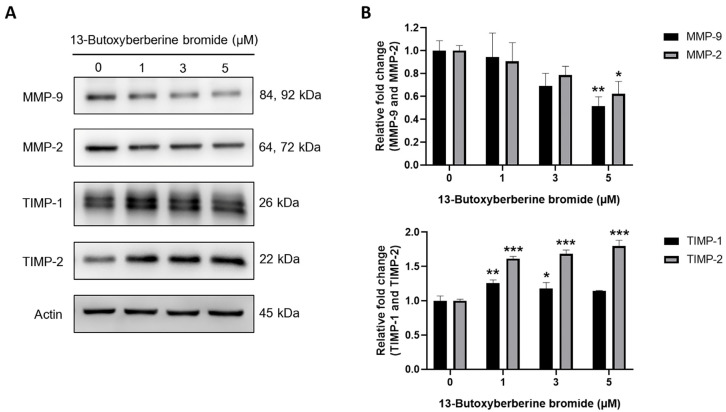
Effect of 13-butoxyberberine bromide on MMPs and TIMPs expressions in A431 cells. Cells were incubated with 13-butoxyberberine bromide. (**A**) Protein expressions of MMP9, MMP2, TIMP-1, and TIMP-2 were examined using Western blot analysis. (**B**) Relative band intensity was measured using Image J software compared to the control group (0.5% DMSO). Data were expressed as mean ± SD (*n* = 3). Actin was used as the loading control. * *p* < 0.05, ** *p* < 0.01, and *** *p* < 0.001; significant difference from the control group.

The effect of 13-butoxyberberine bromide on protein expression levels in A431 cells was evaluated using Western blot analysis. The results indicated that 13-butoxyberberine bromide at 5 µM significantly suppressed MMP-9 and MMP-2 expression compared to the control group (0.5% DMSO). Additionally, the results indicated that the expression levels of TIMP-1 and TIMP-2 were significantly increased upon treatment with 13-butoxyberberine bromide for 24 h (Figure 6), suggesting that 13-butoxyberberine bromide showed inhibition of migration and invasion in A431 cells through MMP suppression.

### 2.7. Effect of 13-Butoxyberberine Bromide on EGFR Signaling Pathway

**Figure 7:**
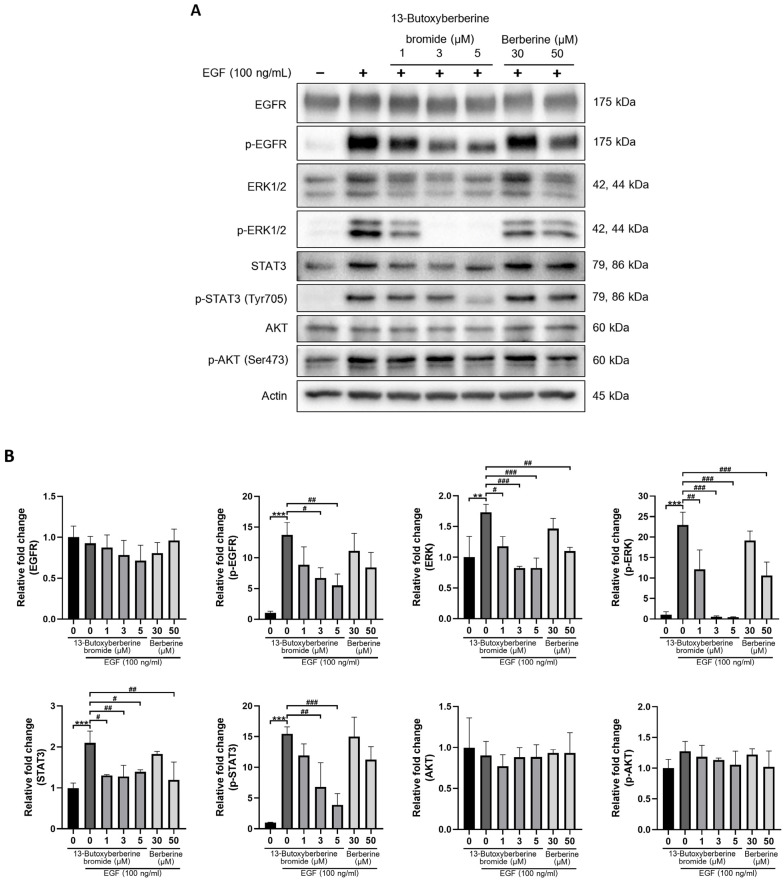
Effect of 13-butoxyberberine bromide on EGFR-mediated ERK, PI3K/Akt, and STAT signaling pathways. Cells were pretreated with 13-butoxyberberine bromide and berberine for 30 min, then induced with EGF for 10 min. (**A**) Protein expressions of EGFR, p-EGFR, ERK, p-ERK, Akt, p-Akt, STAT3, and p-STAT3 were examined using Western blot analysis. (**B**) Relative band intensity was measured using Image J software compared to the control group (0.5% DMSO). Data were expressed as mean ± SD (*n* = 3). Actin was used as the loading control. ** *p* < 0.01 and *** *p* < 0.001; significant difference from the control group without EGF stimulation. ^#^ *p* < 0.05, ^##^ *p* < 0.01, and ^###^ *p* < 0.001; significant difference from the control group with EGF stimulation.

The EGFR-mediated ERK, PI3K/Akt, and STAT signaling pathways play a crucial role in cancer metastasis. To study the target of 13-butoxyberberine bromide, the protein expression of EGFR, ERK, Akt, and STAT3 were estimated using Western blot analysis. A431 cells were pretreated with or without different concentrations of 13-butoxyberberine bromide (1, 3, and 5 µM) and berberine (30 and 50 µM) in 1% FBS medium for 30 min. Then, cells were induced with 100 ng/mL of EGF for 10 min. After that, Western blot analysis was carried out as previously explained. As shown in Figure 7, the expression of p-EGFR, ERK, p-ERK, STAT3, and p-STAT3 were significantly increased in the control group with EGF stimulation (0.5% DMSO + EGF) compared with the control group without EGF (0.5% DMSO only), but there was no significant difference in Akt and p-Akt expression. Interestingly, pretreatment with 13-butoxyberberine bromide significantly inhibited EGF-induced p-EGFR, ERK, p-ERK, STAT3, and p-STAT3 expressions in A431 cells compared to the control group with EGF stimulation. Moreover, 13-butoxyberberine bromide inhibited EGF-induced protein expression at lower concentrations when compared with berberine. This emphasized that 13-butoxyberberine bromide was more effective than berberine. In summary, 13-butoxyberberine bromide suppressed the EGFR-mediated ERK and STAT signaling pathways in A431 cells.

### 2.8. 13-Butoxyberberine Bromide Inhibited EGF-Induced A431 Cell Migration

**Figure 8:**
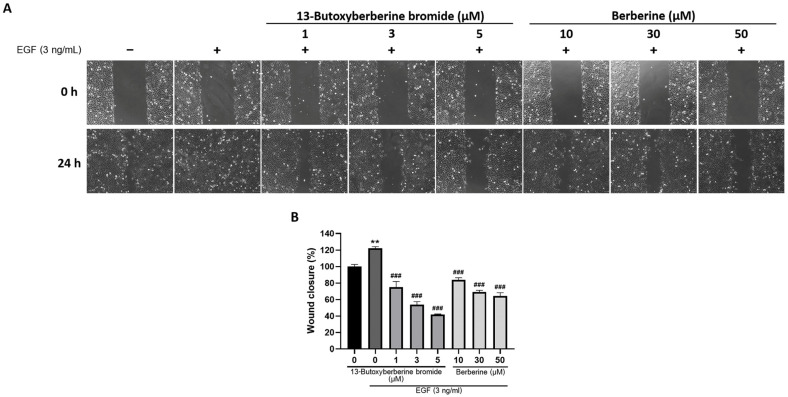
Effect of 13-butoxyberberine bromide and berberine on EGF-induced cell migration in A431 cells. Cells were pretreated with 13-butoxyberberine bromide and berberine for 30 min. Then, cells were induced with EGF before investigating the wound areas. (**A**) The wound distance was observed under an inverted microscope (100× magnification). (**B**) The histogram presents the wound closure percentage after incubation with 13-butoxyberberine bromide and berberine for 24 h. Data were expressed as mean ± SD (*n* = 3). ** *p* < 0.01; significant difference from the control group without EGF stimulation. ^###^ *p* < 0.001; significant difference from the control group with EGF stimulation.

To confirm that 13-butoxyberberine bromide could inhibit EGF-induced A431 cell migration, cells were examined using a wound-healing assay. After scratching, A431 cells were pretreated with or without different concentrations of 13-butoxyberberine bromide (1, 3, and 5 µM) and berberine (10, 30, and 50 µM) in 1% FBS medium for 30 min, then induced with 3 ng/mL of EGF. As shown in Figure 8, the migration of A431 cells was significantly increased in the control group with EGF stimulation (0.5% DMSO + EGF) compared with the control group without EGF (0.5% DMSO only). Pretreatment with 13-butoxyberberine bromide significantly inhibited EGF-induced cell migration compared to the control group with EGF stimulation. In addition, 13-butoxyberberine bromide also inhibited EGF-induced cell migration at lower concentrations when compared with berberine. These results suggested that 13-butoxyberberine bromide could inhibit EGF-induced cell migration of A431 cells.

## 3. Discussion

Metastasis is a complicated process involving the spreading of a primary tumor from its origin and migration to a distant organ, generating a secondary tumor. The metastatic cascades begin from the primary tumor generating new blood or lymphatic vessels; these steps are called angiogenesis and lymphangiogenesis. Then, the primary tumor escapes from the primary organ to the secondary organ. In this step, the cancer cells can invade blood vessels and migrate to the target organs, resulting in cancer cell invasion and migration [25], followed by the tumor cells leaving the circulatory system and colonizing the distant organs [3]. Cancer metastasis is the primary cause of cancer-related death. Therefore, searching for the bioactive compound that could inhibit cancer metastasis is a necessary strategy. Berberine is a plant isoquinoline alkaloid with many pharmacological activities including antioxidant, anti-inflammatory, antibacterial, anticholinergic, antihypertensive and anticancer activities [18,19]. There have been many reports on the anti-metastatic effect of berberine. However, there is limited information on the effect of berberine derivatives on cancer metastasis. In 2020, Zhang C et al. reported the modification of berberine structure for its anticancer effect, mostly concentrated at C-13 and C-9. They suggested that three berberine derivatives could induce cell cycle arrest and apoptosis in colon cancer cells more effectively than berberine [26]. Moreover, Samosorn S et al., 2011, modified the structure of berberine at C-13 with an alkoxy group, obtaining a berberine derivative, 13-butoxyberberine bromide. They found that the 13-butoxyberberine bromide was highly effective against lung, oral, and breast cancer cells, approximately 1.4–546.7 times more potent than berberine, and also more effective than the anticancer drugs ellipticine and doxorubicin. Importantly, this compound was specific to cancer cells and was highly safe for normal cells [24].

Therefore, we investigated the effects of 13-butoxyberberine bromide on the migration, invasion, and underlying mechanisms of skin cancer A431 cells. The cell viability assay indicated that 13-butoxyberberine bromide reduced the cell viability of A431 cells in a dose-dependent manner, with a higher cell growth inhibitory effect than with berberine, which is consistent with Samosorn’s report [24]. For the migration study, we used a wound-healing assay to examine the effects of 13-butoxyberberine bromide on A431 cell migration. This method is appropriate for studying the effects of cell–matrix and cell–cell interactions, and whole-cell migration [27]. This study suggested that the sub-toxic concentrations of 13-butoxyberberine bromide showed the potential to suppress cell migration in skin cancer A431 cells. Moreover, 13-butoxyberberine bromide was more effective in cell migration inhibition than berberine. We confirmed the migration inhibition of 13-butoxyberberine bromide on A431 cells by using a transwell migration assay. This method is popular for studying cell sensitivity to specific chemoattractant substances and their migration across physical barriers [28]. The results indicated that 13-butoxyberberine bromide suppressed A431 cell migration. Furthermore, we investigated the effect of 13-butoxyberberine bromide on A431 cell invasion using a modified transwell chamber, which was modified by coating Matrigel on the bottom of the chamber to mimic ECM. The transwell invasion assay indicated the metastatic process of cancer cells via the ability to digest the ECM proteins, which is important step of cancer cell invasion into blood vessels [29]. The results demonstrated that 13-butoxyberberine bromide suppressed A431 cell invasion. In addition, we examined the effect of 13-butoxyberberine bromide on cell adhesion in A431 cells using an adhesion assay. The adhesion assay is a method of examining a cell’s ability to adhere to ECM proteins or other cells, based on the colorimetric detection of bound cells using a phase-contrast microscope [30]. We found that 13-butoxyberberine bromide also reduced cell adhesion ability in A431 cells, which can be observed from the reduction in cell elongation in a Matrigel-coated plate. A previous study reported that berberine reduced cell viability and inhibited the migration and invasion of human melanoma A375.S2 cells [31], which is consistent with our results.

Cancer cell invasion and migration are regulated by many molecules of proteins, especially the MMP and TIMP proteins. MMPs are a group of proteolytic enzymes that play key roles in cancer cell invasion and metastasis, whereas TIMPs are endogenous inhibitors for controlling the activity of MMPs [32]. Active MMPs play vital roles in cancer progression by triggering ECM degradation and inducing cancer migration and invasion [33]. Thus, the increase in MMPs expression is directly related to cancer cell invasion, metastasis, and angiogenesis [34]. Many previous studies reported that berberine suppressed the expression of MMPs in mammalian cells such as inflamed skin cells [35] and breast cancer cells [36]. In this study, we found that 13-butoxyberberine bromide decreased MMP-9 and MMP-2 expression and increased TIMP-1 and TIMP-2 expression. A previous study suggested that berberine suppressed the migration and invasion of A431 cells [37] and also inhibited human melanoma A375.S2 cell migration and invasion via inhibition of MMP-9 [31]. These reports correlated with our results.

Several studies reported that the EGFR signaling pathway regulated cancer metastasis [11] and MMP expression [12,13]. In this study, we examined the expression of proteins in the EGFR signaling pathway in EGFR-overexpressed A431 cells. We found that pretreatment with 13-butoxyberberine bromide significantly inhibited EGF-induced p-EGFR, ERK, p-ERK, STAT3, and p-STAT3 expression in A431 cells. It has been reported that berberine suppressed EGFR/STAT3 signaling pathways in gastric cancer cell lines [38]. Moreover, berberine-down-regulated EGFR activation stimulated by EGF led to a reduction in phosphorylated EGFR expression in prostate cancer PC-3 cells [39], which supported our findings. Our results showed no significant increase in Akt and p-Akt expression after EGF induction, which correlated with a previous study that reported the level of p-Akt in A431 cells was relatively stable after EGF induction [40]. Although there was no significant difference, p-Akt expression tended to increase after EGF induction and decrease with pretreatment with 13-butoxyberberine bromide. Moreover, 13-butoxyberberine bromide inhibited EGF-induced protein expressions at lower concentrations when compared with berberine. Furthermore, we confirmed that 13-butoxyberberine bromide could inhibit EGF-induced A431 cell migration at lower concentrations when compared with berberine. This emphasized that 13-butoxyberberine bromide was more effective than berberine. Thus, our study suggested that 13-butoxyberberine bromide inhibited migration and invasion in A431 cells by down-regulated MMP-9 and MMP-2 expression and up-regulated TIMP-1 and TIMP-2 expression. In addition, 13-butoxyberberine bromide inhibited cell migration and the EGFR signaling pathway in EGF-induced A431 cells.

## 4. Materials and Methods

### 4.1. Cell Culture

Human epidermoid carcinoma cell line A431 and human keratinocyte cell line HaCat were obtained from the American Type Culture Collection (ATCC; Manassas, VA, USA). Cells were maintained in Dulbecco’s modified Eagle’s medium (DMEM) (Invitrogen Life Science, Waltham, MA, USA) supplemented with 10% fetal bovine serum (FBS) (GE Healthcare, Chalfont, UK), penicillin, and streptomycin (PAA Laboratories, Pasching, Austria). The cells were cultured in humidified incubators at 37 ºC and 5% CO_2_.

### 4.2. Chemical Reagents

**Figure 9:**
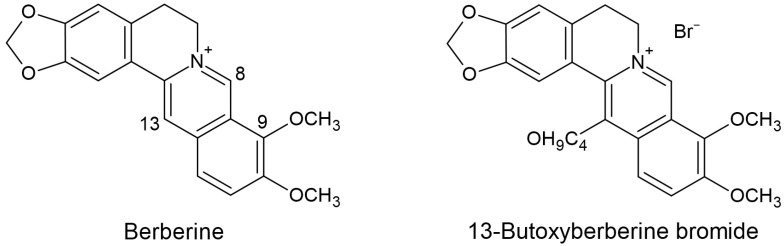
The chemical structures of berberine and 13-butoxyberberine bromide.

Berberine and 13-butoxyberberine bromide (berberine derivative) (Figure 9) were obtained from Assoc. Prof. Siritron Samosorn, Department of Chemistry, Faculty of Science, Srinakharinwirot University, Thailand. The compounds were dissolved in DMSO.

### 4.3. Cell Viability Assay

Cell viability was examined using an MTT assay. Cells at 1 × 10^4^ cells/well were cultured for 24 h. Then, cells were treated with berberine and 13-butoxyberberine bromide at various concentrations, whereas the control group was treated with 0.5% DMSO for 24 h. After treatment, 0.5 mg/mL of MTT solution was added. The formazan crystals were dissolved with DMSO and measured using a spectrophotometer at 570 nm. The percentage of cell survival was calculated and sub-toxic concentrations (≥80% cell survival) of berberine and 13-butoxyberberine bromide were chosen for further experiments.

### 4.4. Wound-Healing Assay

Cells were cultured at 50 × 10^4^ cells/well, then the cell monolayer was wounded by scratching with a plastic tip and washed with 1× phosphate-buffered saline (PBS). The cells were incubated with or without different concentrations of berberine and 13-butoxyberberine bromide. The wound distance was measured and calculated as the percentage of cell migration, as compared to the untreated control (0.5% DMSO).

### 4.5. Transwell Invasion and Migration Assay

The ability of cells to invade the Matrigel-coated membrane filter was measured in a transwell chamber assay. The transwell chambers (Merck Millipore Corp., Darmstadt, Germany) were inserted into a 24-well plate and coated with 0.4 mg/mL Matrigel. The cells in the serum-free medium with or without various concentrations of 13-butoxyberberine bromide were cultured in the upper chambers. The lower chamber was filled with 10% FBS medium, which served as a chemoattractant, and incubated for 24 h. After incubation, the non-invaded cells were removed using a cotton swab and the invaded cells on the lower surface of the membrane were fixed with methanol and stained with 0.5% crystal violet. The cells were photographed, and the cell invasion percentage was calculated as compared to the untreated control (0.5% DMSO). For the migration assay, the cells were treated the same as the invasion assay, but without membrane-coating with Matrigel.

### 4.6. Adhesion Assay

Cells were cultured at 30 × 10^4^ cells/dish for 24 h, then incubated with or without various concentrations of 13-butoxyberberine bromide for 24 h. After that, the cells were harvested and seeded in a Matrigel-coated plate (1 × 10^4^ cells/well) for 1 h. The non-adhering cells were detached and rinsed with 1X PBS. The adhering cells were examined using an MTT assay. The cell adhesion percentage was calculated as compared with the untreated control (0.5% DMSO).

### 4.7. Western Blot Analysis

Cells were seeded in a 6-well plate and incubated with or without different concentrations of 13-butoxyberberine bromide for 24 h. After that, the cells were harvested and lysed with RIPA buffer (50 mM Tris-HCl, pH 7.5, 5 mM EDTA, 250 mM NaCl, 0.5% Triton X-100) containing a complete mini protease inhibitor cocktail (Roche Diagnostics GmbH, Mannheim, Germany). The concentration of protein was measured using a Bradford assay. Protein samples were separated using 8–10% SDS-PAGE and transferred to polyvinylidene difluoride (PVDF) membranes (Merck Millipore Corp., Merck KGaA, Darmstadt, Germany). The membranes were blocked with 5% BSA for 1 h. The membranes were incubated with primary antibodies and the horseradish peroxidase (HRP)-conjugated secondary antibody (Cell Signaling Technology, Beverly, MA, USA). The signals were examined using enhanced chemiluminescence (ECL) (Merck Millipore Corp., Merck KGaA, Cell Signaling Technology, Beverly, MA) and exposed using a CCD camera.

### 4.8. Statistical Analysis

All data were expressed as mean ± standard deviation (SD) of three independent experiments in triplicate. The statistical significance was analyzed with one-way ANOVA (Turkey’s multiple comparisons test) using the GraphPad Prism 8.0 software (version 8.0, Boston, MA, USA).

## 5. Conclusions

Our findings demonstrated that 13-butoxyberberine bromide inhibited A431 cell migration and invasion by down-regulated MMP-9 and MMP-2 expression and up-regulated TIMP-1 and TIMP-2 expression. In addition, 13-butoxyberberine bromide inhibited cell migration and the EGFR signaling pathway in EGF-induced A431 cells. Moreover, 13-butoxyberberine bromide was more effective than berberine, suggesting that 13-butoxyberberine bromide could be a candidate anti-metastatic agent for cancer treatment. Our findings may be helpful in the development of 13-butoxyberberine bromide as an effective anti-metastatic agent for future clinical applications.

## Data Availability

The datasets analyzed during the current study are available from the corresponding author on reasonable request.

## Sample Availability

Samples of the compounds are available from the authors.

## References

[1] Guan X. (2015). Cancer metastases: Challenges and opportunities. Acta Pharm. Sin. B.

[2] Seyfried T.N., Huysentruyt L.C. (2013). On the origin of cancer metastasis. Crit. Rev. Oncog..

[3] van Zijl F., Krupitza G., Mikulits W. (2011). Initial steps of metastasis: Cell invasion and endothelial transmigration. Mutat. Res..

[4] Lynch C.C., Matrisian L.M. (2002). Matrix metalloproteinases in tumor-host cell communication. Differentiation.

[5] Heldin C.H., Landström M., Moustakas A. (2009). Mechanism of TGF-beta signaling to growth arrest, apoptosis, and epithelial-mesenchymal transition. Curr. Opin. Cell Biol..

[6] Yan C., Boyd D.D. (2007). Regulation of matrix metalloproteinase gene expression. J. Cell. Physiol..

[7] Tomas A., Futter C.E., Eden E.R. (2014). EGF receptor trafficking: Consequences for signaling and cancer. Trends Cell Biol..

[8] Singh M., Jadhav H.R. (2018). Targeting non-small cell lung cancer with small-molecule EGFR tyrosine kinase inhibitors. Drug Discov. Today.

[9] Siena S., Sartore-Bianchi A., Di Nicolantonio F., Balfour J., Bardelli A. (2009). Biomarkers predicting clinical outcome of epidermal growth factor receptor-targeted therapy in metastatic colorectal cancer. J. Natl. Cancer Inst..

[10] Wieduwilt M.J., Moasser M.M. (2008). The epidermal growth factor receptor family: Biology driving targeted therapeutics. Cell. Mol. Life Sci..

[11] Shen T., Guo Q. (2019). EGFR signaling pathway occupies an important position in cancer-related downstream signaling pathways of Pyk2. Cell Biol. Int..

[12] Sternlicht M.D., Werb Z. (2001). How matrix metalloproteinases regulate cell behavior. Annu. Rev. Cell Dev. Biol..

[13] Anand M., Van Meter T.E., Fillmore H.L. (2011). Epidermal growth factor induces matrix metalloproteinase-1 (MMP-1) expression and invasion in glioma cell lines via the MAPK pathway. J. Neurooncol..

[14] Zhao H., Halicka H.D., Li J., Darzynkiewicz Z. (2013). Berberine suppresses gero-conversion from cell cycle arrest to senescence. Aging.

[15] Xia L.M., Luo M.H. (2015). Study progress of berberine for treating cardiovascular disease. Chronic Dis. Transl. Med..

[16] Lao-ong T., Chatuphonprasert W., Nemoto N., Jarukamjorn K. (2012). Alteration of hepatic glutathione peroxidase and superoxide dismutase expression in streptozotocin-induced diabetic mice by berberine. Pharm. Biol..

[17] Kumar A., Ekavali, Chopra K., Mukherjee M., Pottabathini R., Dhull D.K. (2015). Current knowledge and pharmacological profile of berberine: An update. Eur. J. Pharmacol..

[18] Liu W., Liu P., Tao S., Deng Y., Li X., Lan T., Zhang X., Guo F., Huang W., Chen F. (2008). Berberine inhibits aldose reductase and oxidative stress in rat mesangial cells cultured under high glucose. Arch. Biochem. Biophys..

[19] Singh S., Pathak N., Fatima E., Negi A.S. (2021). Plant isoquinoline alkaloids: Advances in the chemistry and biology of berberine. Eur. J. Med. Chem..

[20] Naveen C.R., Gaikwad S., Agrawal-Rajput R. (2016). Berberine induces neuronal differentiation through inhibition of cancer stemness and epithelial-mesenchymal transition in neuroblastoma cells. Phytomedicine.

[21] Kou Y., Li L., Li H., Tan Y., Li B., Wang K., Du B. (2016). Berberine suppressed epithelial mesenchymal transition through cross-talk regulation of PI3K/AKT and RARα/RARβ in melanoma cells. Biochem. Biophys. Res. Commun..

[22] Kim H.S., Kim M.J., Kim E.J., Yang Y., Lee M.S., Lim J.S. (2012). Berberine-induced AMPK activation inhibits the metastatic potential of melanoma cells via reduction of ERK activity and COX-2 protein expression. Biochem. Pharmacol..

[23] Wang L., Cao H., Lu N., Liu L., Wang B., Hu T., Israel D.A., Peek R.M., Polk D.B., Yan F. (2013). Berberine inhibits proliferation and down-regulates epidermal growth factor receptor through activation of Cbl in colon tumor cells. PLoS ONE.

[24] Samosorn S., Tanwirat B., Suksamrarn A. (2011). Anticancer activity of 13-alkoxy berberine derivatives. Thai Patent.

[25] Chu S.C., Yu C.C., Hsu L.S., Chen K.S., Su M.Y., Chen P.N. (2014). Berberine reverses epithelial-to-mesenchymal transition and inhibits metastasis and tumor-induced angiogenesis in human cervical cancer cells. Mol. Pharmacol..

[26] Zhang C., Sheng J., Li G., Zhao L., Wang Y., Yang W., Yao X., Sun L., Zhang Z., Cui R. (2019). Effects of berberine and its derivatives on cancer: A systems pharmacology review. Front. Pharmacol..

[27] Rodriguez L.G., Wu X., Guan J.L. (2005). Wound-healing assay. Methods Mol. Biol..

[28] Justus C.R., Leffler N., Ruiz-Echevarria M., Yang L.V. (2014). In vitro cell migration and invasion assays. J. Vis. Exp..

[29] Liew K., Yong P.V., Lim Y.M., Navaratnam V., Ho A.S. (2014). 2-Methoxy-1,4-Naphthoquinone (MNQ) suppresses the invasion and migration of a human metastatic breast cancer cell line (MDA-MB-231). Toxicol. In Vitro.

[30] Humphries M.J. (2001). Cell adhesion assays. Mol. Biotechnol..

[31] Liu J.F., Lai K.C., Peng S.F., Maraming P., Huang Y.P., Huang A.C., Chueh F.S., Huang W.W., Chung J.G. (2018). Berberine inhibits human melanoma A375.S2 cell migration and invasion via affecting the FAK, uPA, and NF-κB signaling pathways and inhibits PLX4032 resistant A375.S2 cell migration in vitro. Molecules.

[32] Tayeh M., Nilwarangoon S., Mahabusarakum W., Watanapokasin R. (2017). Anti-metastatic effect of rhodomyrtone from Rhodomyrtus tomentosa on human skin cancer cells. Int. J. Oncol..

[33] Naglich J.G., Jure-Kunkel M., Gupta E., Fargnoli J., Henderson A.J., Lewin A.C., Talbott R., Baxter A., Bird J., Savopoulos R. (2001). Inhibition of angiogenesis and metastasis in two murine models by the matrix metalloproteinase inhibitor, BMS-275291. Cancer Res..

[34] Peng P.L., Hsieh Y.S., Wang C.J., Hsu J.L., Chou F.P. (2006). Inhibitory effect of berberine on the invasion of human lung cancer cells via decreased productions of urokinase-plasminogen activator and matrix metalloproteinase-2. Toxicol. Appl. Pharmacol..

[35] Kim S., Kim Y., Kim J.E., Cho K.H., Chung J.H. (2008). Berberine inhibits TPA-induced MMP-9 and IL-6 expression in normal human keratinocytes. Phytomedicine.

[36] Kim S., Han J., Lee S.K., Choi M.Y., Kim J., Lee J., Jung S.P., Kim J.S., Kim J.H., Choe J.H. (2012). Berberine suppresses the TPA-induced MMP-1 and MMP-9 expressions through the inhibition of PKC-α in breast cancer cells. J. Surg. Res..

[37] Li D.X., Zhang J., Zhang Y., Zhao P.W., Yang L.M. (2015). Inhibitory effect of berberine on human skin squamous cell carcinoma A431 cells. Genet. Mol. Res..

[38] Wang J., Yang S., Cai X., Dong J., Chen Z., Wang R., Zhang S., Cao H., Lu D., Jin T. (2016). Berberine inhibits EGFR signaling and enhances the antitumor effects of EGFR inhibitors in gastric cancer. Oncotarget.

[39] Huang Z.H., Zheng H.F., Wang W.L., Wang Y., Zhong L.F., Wu J.L., Li Q.X. (2015). Berberine targets epidermal growth factor receptor signaling to suppress prostate cancer proliferation in vitro. Mol. Med. Rep..

[40] Magi S., Saeki Y., Kasamatsu M., Tashiro E., Imoto M. (2014). Chemical genomic-based pathway analyses for epidermal growth factor-mediated signaling in migrating cancer cells. PLoS ONE.

